# Ancestral European roots of *Helicobacter pylori *in India

**DOI:** 10.1186/1471-2164-8-184

**Published:** 2007-06-20

**Authors:** S Manjulata Devi, Irshad Ahmed, Paolo Francalacci, M Abid Hussain, Yusuf Akhter, Ayesha Alvi, Leonardo A Sechi, Francis Mégraud, Niyaz Ahmed

**Affiliations:** 1 Pathogen Evolution Group, Centre for DNA Fingerprinting and Diagnostics, Hyderabad, India; 2 Centre for Liver Research and Diagnostics, Deccan College of Medical Sciences and allied Hospitals, Hyderabad, India; 3 Department of Microbiology, Shri Shivaji College of Arts, Commerce and Science (SGB Amravati University), Akola, MS, India; 4 Dipartimento di Zoologia e Genetica Evoluzionistica, University of Sassari, Sassari, Italy; 5 ISOGEM Collaborative Network on Genetics of Helicobacters (The International Society for Genomic and Evolutionary Microbiology, University of Sassari, Sassari, Italy); 6 Dipartimento de Scienze Biomediche, University of Sassari, Sassari, Italy; 7 INSERM U853 and Centre National de Référence des Campylobacters et Hélicobacters, Laboratoire de Bactériologie, Université Victor Segalen Bordeaux 2, France

## Abstract

**Background:**

The human gastric pathogen *Helicobacter pylori *is co-evolved with its host and therefore, origins and expansion of multiple populations and sub populations of *H. pylori *mirror ancient human migrations. Ancestral origins of *H. pylori *in the vast Indian subcontinent are debatable. It is not clear how different waves of human migrations in South Asia shaped the population structure of *H. pylori*. We tried to address these issues through mapping genetic origins of present day *H. pylori *in India and their genomic comparison with hundreds of isolates from different geographic regions.

**Results:**

We attempted to dissect genetic identity of strains by multilocus sequence typing (MLST) of the 7 housekeeping genes (*atp*A, *efp*, *ure*I, *ppa*, *mut*Y, *trp*C, *yph*C) and phylogeographic analysis of haplotypes using MEGA and NETWORK software while incorporating DNA sequences and genotyping data of whole *cag *pathogenicity-islands (*cag*PAI). The distribution of *cag*PAI genes within these strains was analyzed by using PCR and the geographic type of *cag*A phosphorylation motif EPIYA was determined by gene sequencing. All the isolates analyzed revealed European ancestry and belonged to *H. pylori *sub-population, hpEurope. The *cag*PAI harbored by Indian strains revealed European features upon PCR based analysis and whole PAI sequencing.

**Conclusion:**

These observations suggest that *H. pylori *strains in India share ancestral origins with their European counterparts. Further, non-existence of other sub-populations such as hpAfrica and hpEastAsia, at least in our collection of isolates, suggest that the hpEurope strains enjoyed a special fitness advantage in Indian stomachs to out-compete any endogenous strains. These results also might support hypotheses related to gene flow in India through Indo-Aryans and arrival of Neolithic practices and languages from the Fertile Crescent.

## Background

Analysis of genetic diversity in microorganisms normally reflects patterns of their own evolution although it is very rare that this can portray their hosts' evolution. Co-evolution between host and pathogens can be explained only if pathogens are not horizontally transmitted, and this supports a possible phylogenetic and evolutionary parallel of the host and pathogens. Sadly, in many cases frequent horizontal transmission separates the evolution of the bacterium from that of the host. However, for some pathogens, such as *H. pylori *[1-3], and JC viruses [4], transmission is faithfully restricted to families within specific communities. This phenomenon has in recent times provided evidence regarding patterns of human migration [2,4,5] in different continents.

The human gastric pathogen *H. pylori *is presumed to have co-evolved with its host [6] and established itself in the human stomach possibly millions of years ago [7]. It has been recognized recently as a reliable biological marker of host-pathogen co-evolution and ancient human migration based on sequence variation in select gene loci. *H. pylori *are genetically diverse to the extreme, providing about 1,400 informative sites within 3.5 to 4.5 kb of sequence from housekeeping genes, and their global genetic structure based on such sequence-haplotypes parallels that of humans [2]. Moreover, epidemiological studies have shown that transmission occurs predominantly within families [8-11]. *H. pylori *therefore, could provide a window into human origins and migration [1,3] and the impact of religions and social systems on stratification of human ethnic groups [12].

A landmark study based on PCR based DNA motif analysis proposed that *H. pylori *jumped recently from animals to humans and, therefore, the acquisition of *H. pylori *by humans may be a recent phenomenon [13]. This study has been the basis for the idea of *'H. pylori *free New World' [13]. However, several independent studies based on large-scale analyses of candidate gene polymorphisms contrasted the idea of recent acquisition and suggest that *H. pylori *might have co-evolved with humans [1,6,14].

Using the same set of Peruvian isolates described earlier by Kersulyte *et al. *[13], Devi *et al*. [3], from our group have suggested that the genetic make up of south American isolates could be an admixture of ancestral and modern lineages of *H. pylori*. They clearly highlighted presence of ancestral *H. pylori *in Peruvians that possibly survived influxes of Spanish strains from Iberian expansions in Peru about 500 years ago. Also, according to this study, the survival advantage of indigenous strains was possibly due to the acquisition of western type *cag*PAIs from newly arrived Spanish strains.

Previous genotyping studies on Indian isolates have largely targeted molecular epidemiological issues. However, Wirth *et al. *[12], for the first time, using *H. pylori *genotypes, addressed issues such as impact of two different religions and societal systems on stratification of human ethnic groups [12] in the remotest north eastern Ladakh area of India. In view of intriguing ideas on ancient origin of *H. pylori*, and the fact that ancient origins and arrival of *H. pylori *are hardly known in the context of the vast South Asian continent, additional evidences based on strains from different geographical regions of Asia are clearly needed.

In this study, we attempted to unravel population genetic structure and gene pool diversity of Indian isolates of *H. pylori *from culturally and linguistically diverse ethnic Indians. The main objective behinds the study has been to explore genetic features of the strains that might explain their ancestral origin and might help reconstruct different waves of pre-historic human migration in India. We also looked if it is possible to link some of the native strains to their ancestors in West Asia, Eurasia or Europe.

## Results

### DNA isolates, diagnostic PCR and epidemiological genotyping

**Figure 1:**
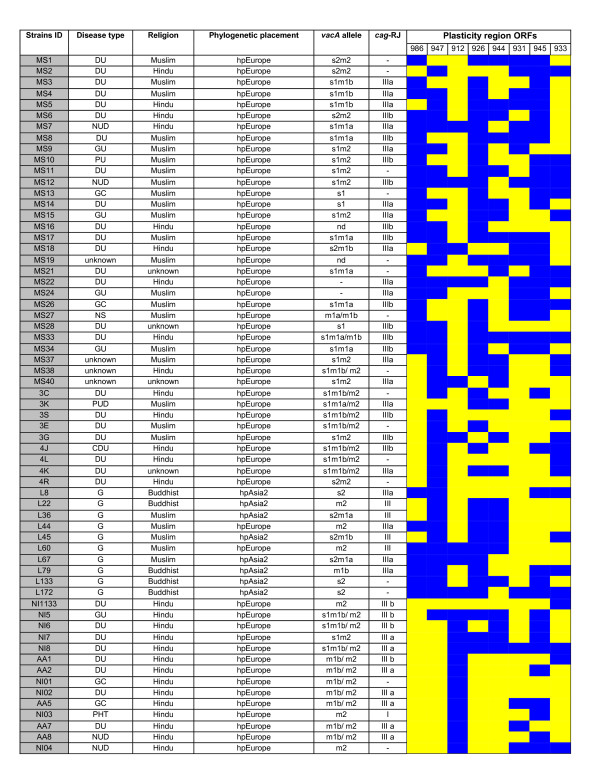
Detailed characteristics of Indian *H. pylori *isolates used in the study. [Yellow, region amplified or present; Blue, region absent or rearranged; -, region failed to amplify].

DNA quality and purity was confirmed by agarose gel electrophoresis and diagnostic PCRs revealed presence of *cag*A, *ice*A, *vac*A, *glm*M, *bab*B and *oip*A genes in all the Indian isolates we tested. The molecular epidemiological features of all the 63 strains we analyzed have been elaborated in Figure 1. Our isolates were quite diverse with respect to the plasticity region ORFs that we analyzed and no specific signature was seen dominant as regards to the arrangement or rearrangement of these ORFs. This validated that all the isolates that we looked at were in fact independent and did not represent any derivatives of clonal evolution.

Specific primers amplifying different alleles (see methods section) were used to analyze the *vac*A allelic diversity. The sizes of the amplified products for *vacA *s1 and *vacA *s2 were 259 bp and 286 bp respectively. Of the 63 isolates analyzed, the s1 allele was detected in 33 (52.3%) and the s2 allele type was detected in 11 (17.4%) strains. The m1 variant was detected in 34 (53.9%) and the m2 variant in 37 (58.7%). The highly toxigenic *vac*A allele combination s1m1 was found to be dominant (33.3%) as compared to other *vac*A allele types. The *vac*A genotype s1m2 was detected in 9 isolates (14.2%) whereas *vacA *s2m1 and *vacA *s2m2 genotypes were detected in 4 isolates (6.3%) each. Not all the isolates yielded full *vac*A amplicons, as regions of *vac*A gene, in particular, the signal region posed difficulty in amplification. This is a very common phenomenon observed in *H. pylori *owing to frequent recombination. The *vacA *alleles have been shown to differ in frequency and type among East Asian isolates [15], for instance, s1c is the predominant signal sequence allele among East Asian isolates [16]. Typically, the *vac*A s1c was found to be completely absent in the Indian isolates.

### Multilocus sequence analysis

**Figure 2:**
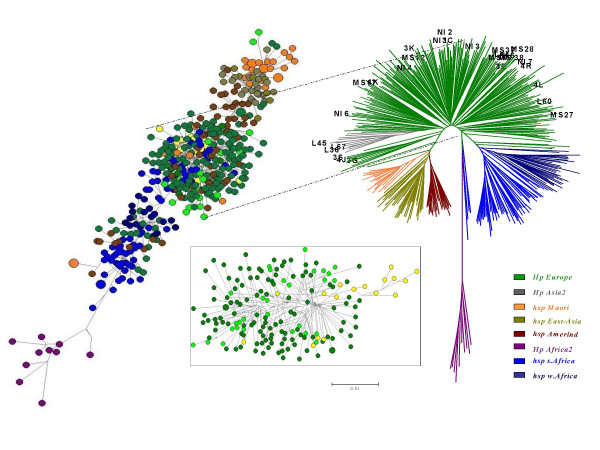
Neighbor joining tree (Kimura 2-parameter) (right) showing the global population structure of *H. pylori *wherein Indian isolates are highlighted. The phylogenetic tree was based on a total of 23 sequence records of South and North Indian isolates while incorporating ~400 other sequence records from pubMLST database representing different *H. pylori *populations and sub populations in the world. The population genetic structure was investigated by determining the multilocus haplotypes based on concatenated sequences of seven unlinked housekeeping genes that are scattered around the *H. pylori *chromosome. Individual isolates were assigned to bacterial populations called hpEastAsia (sub-populations: hspEAsia, hspMaori, hspAmerind), hpEurope, hpAfrica1 (hspSAfrica, hspWAfrica), hpAsia2 and hpAfrica2 [11]. Representatives from each of these (sub)-populations were chosen for subsequent analysis of the *cag*PAI. Isolates from the population hpAfrica2 do not contain *cag*PAI. Phylogenetic relationships were also estimated through NETWORK analysis (left) based on 665 mutating positions that revealed the co-evolution of the *H. pylori *genome. The Ladakhi (yellow) and other Indian (light green) lineages were more clearly discerned within the European (dark green) cluster (centre box), when analyses based on the remaining 650 mutating positions were performed. For the Neighbor-joining tree (right), the bootstrap values of the interior branches as calculated in MEGA, were significantly high to indicate the correct topology of the branches within the clades.

We report that almost all of the *H. pylori *strains from India share significant homology to the members of sub-population hpEurope. A total of 33 MLST profiles based on DNA sequence of a concatenated multigene comprising of 7 individual gene loci (*atpA, efp, mutY, ppa, trpC, ureI *and *yphC*) were generated from Indian isolates. Data comprising of these MLST profiles were subjected to comparative genomic analysis with ~400 other *H. pylori *sequences from different geographical and ethnic groups [11]. Such analyses upon construction of a neighbor-joining tree in MEGA 3.1 software using Kimura-2 parameter revealed clear geographic distribution of various *H. pylori *populations and sub-populations, essentially in accordance with the previous results [1,3,17]. All the Indian isolates from North and South India and 2 of them from Ladakh clustered under hpEurope. Seventeen Ladakhi isolates clustered tightly to form a separate branch, hpAsia2. Results of MLST analysis in MEGA3.1 were successfully reproduced using NETWORK based phylogeny, which revealed similar acquaintances for *H. pylori *in India. Mirroring the spread of human populations from Africa, our network analysis suggests the co-evolution of *H. pylori *with *Homo sapiens*, as also suggested recently [6]. Both the domains of the Network tree based on 650 (data not shown) and 665 (Figure 2, left) mutating positions clearly separated African from non-African sequences. The second domain seemed to harbor higher phylogenetic information, since the resulting graph is more clearly structured, with a more accurate separation among European, Amerindian, Asian and Australasian lineages. The Indian *H. pylori *sequences were clustered within the European portion of the network, wherein the first domain identifies a separate branch, encompassing the majority of the Ladakhi samples, as a distinct sub-population of hpAsia2 within the European variability, and remarking the isolation of the human host population. However, many of the Ladakhi Muslim samples clustered in hpEurope and revealed a significant sequence similarity to the mainland Indian samples. These results are in agreement with previous studies on the hypervariable region of human mitochondrial DNA that showed the common origin of European and Indian populations [18] and the relative homogeneity of Indian populations regardless of their ethnic and linguistic affiliation [19].

### Analysis of the *cag*PAI and its Right Junction (RJ) motifs

**Figure 3:**
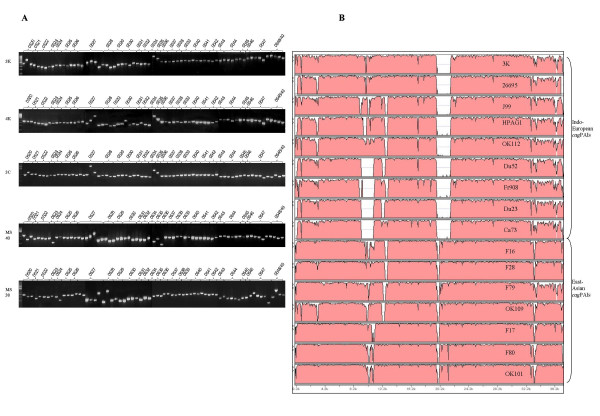
Comparative genomic analysis of the *cag*PAIs from Indian isolates. A) PCR based analysis of the complete *cag*PAI of 5 representative hpEurope Isolates: 3K, 4K, 3C, MS40 and MS38 from India. Overlapping PCR primers amplified the whole *cag*PAI indicating the intactness of the PAI in these isolates. B) Global pair-wise alignments of whole *cag*PAI sequences of different *H. pylori *isolates were generated by VISTA using default parameters [47]. The OK129 genome was taken as the base sequence (not shown) and rest of the sequences were aligned against it. The X-axis denotes length of the sequence under consideration and the Y-axis conveys homology in % with the base genome sequence). The Indian hpEurope isolate, 3K was aligned with other whole *cag*PAI sequences from GenBank along with the *cag*PAIs of HP 26695, HPJ99 and HPAG1. The accession numbers for the public domain sequences of the *cag*PAIs from Europe [9] and Japan [49] that we used in our analyses, were as follows – Ca73 (AY330638 and AY330639), Du23:2 (AY330643 and AY330644), Du52:2 (AY330640, AY330641 and AY330642), F80 (AB120421), OK112 (AB120425), F16 (AB120416), F17 (AB120417), F28 (AB120418), F79 (AB120420), OK101 (AB120422), OK109 (AB120424). Sequence of the French isolate, Fr 908 was determined in this study (EF195721). While the *cag*PAI sequence of the Indian isolate 3K (hpEurope) was found to be genetically highly similar to and aligning closely with the 26695 sequence, it also revealed significant sequence similarities with other isolates of European origins (that harbor Western type of *cag *EPIYA sequences) such as HPAG1, OK112, Du52, Du23, Ca73, J99 and Fr908. It was however largely unrelated to the East Asian like isolates (mainly harboring Asian type *cag *EPIYA sequences) such as F16, F28, F79, OK109, F17, OK101 and F80.

Overlapping primer amplification to span entire *cag*PAI worked reproducibly with our isolates; Figure 3(A) reveals complete PCR output for the ~38 kb *cag*PAI region in 5 representative strains MS38, MS40, 3K, 4K and 3C. All the constituent genes of the PAI were successfully amplified for all the Indian isolates studied. To get more insights into composition and arrangement of the gene loci within the PAI, complete sequencing of the *cag*PAI of isolate 3K was performed. This isolate was from a patient with peptic ulcer disease (PUD) from South India. The size of complete *cag*PAI of this isolate was 36,876 bp with a G+C content of 35.9. The sequence composition and gene order in the *cag*PAI of 3K was compared to those of the three completely sequenced strains 26695, J99 and HPAG1 which revealed some minor differences such as fused HP0521 and HP0522 genes due to the deletion of a single nucleotide at the 3' end of HP0521. Similarly single or dinucleotide differences were observed in the *cagX *(HP0528), *cagN *(HP0538) and *cagE *(HP0544) and most of these insertions and deletions were observed in the intergenic regions. Broadly, the *cag*PAI genes were very conserved as regards to the amino acid sequences when compared with at least 15 different publicly available *cag*PAI sequences.

*cag*-RJ (the extreme right junction of the *cag*PAI, between 3' end of the *cag*A gene and the start of the glutamate racemase – *glr*) was studied for our 63 isolates where 99% isolates harbored type III motif. A total of 47 of 63 strains (75%) gave positive PCR results for *cag*-RJ (Figure 1). The type III motif was found in 27 of 39 South Indian isolates and 20 of 24 North Indian isolates. It is noteworthy that *cag*-RJ typeIII motifs are genetically close to European type I motifs probably due to an ancient insertion event, followed by recombinational scrambling among type I and III lineages [13]. We did not find in our Indian isolates any type II motifs, which constitute a signature characteristic of East Asian gene pool.

### Genetic relationship of Indian isolates based on *cag*A and whole *cag*PAI sequences

A full-length *cag*PAI sequence based alignment was constructed using the Indo-European type 3K and Afro-European type Fr908 (French patient isolate) sequences determined in this study, along with 15 different whole *cag*PAI sequence from GenBank: Ca73, Du23: 2, Du52: 2, F16, F17, F28, F79, F80, OK101, OK109, OK112, OK129, 26695, J99 and HPAG1. Our South Indian isolate, 3K, was found to be aligning with the Western *cag*PAI sequences (Figure 3B).

**Figure 4:**
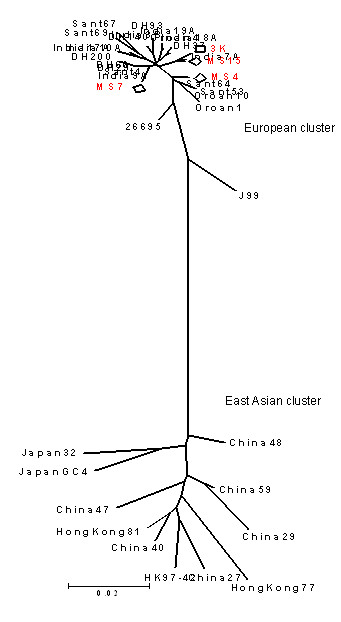
Phylogenetic tree based on the 5' end sequence of the *cag*A (an informative 219 bp segment of *cag*A was used to align sequences from unrelated isolates) suggests possible common origins for isolates from ethnic Indians and the tribal. Representative Indian genotypes (3K, MS4, Ms7 and MS15) based on this 219 bp sequence clustered tightly with previously determined genotypes of strains obtained from ethnic Bengalis [India3B (AF202219), India7A (AF202220), India9A (AF202221), India10A (AF202222), India17A (AF202223), India18A (AF202224), India19A (AF202225), DH140 (AY169293), DH200 (AY169294), DH29 (AY169295), DH37 (AY169296), DH60 (AY169297), DH93 (AY169298)] and Santhal and Oraon tribals [Sant4 (AY162446), Sant53 (AY162447), Sant64 (AY162448), Sant67 (AY162449), Sant69 (AY162450), Oraon1 (AY162451), Oraon10 (AY162452), Oraon4 (AY162453)] [20]. All the East Asian strains [China27 (AJ252979), China29 (AJ252980), China40 (AJ252982), China48 (AJ252983), China47 (AJ252985), China59 (AJ252986), Hongkong77 (AF198485), Hongkong81 (AF198486), Hongkong97-42 (AF239733), Japan GC4 (AF198484), Japan32 (AJ239726)], however, clustered together and formed a separate cluster.

We examined relatedness of the *cag*A gene sequences of tribal isolates from India to the mainstream Indian isolates and the European isolates by analyzing a 219 bp informative fragment near the 5' end of *cag*A which usually distinguishes the European and the East Asian strains [20]. Comparative sequence analysis was used to construct phylogenetic relationship in MEGA3.1. All the sequence records corresponding to the isolates of Santhal and Oraon tribals revealed homologies to the main stream Indian strains from Hyderabad, Lucknow and Bengal and also to all the representative European strains. These tribal isolates did not cluster with East Asian strains (Figure 4).

This makes it clear that the *cag*PAI of Indian strains is a completely evolved one and probably was acquired from a European source, well before the arrival of *H. pylori *in India. This is also evident from the fact that the Indian strains, though of a European descent, do not share characteristic features of Asian *cag*PAIs.

## Discussion

Although the Indian peninsula has seen many different waves of population migration [21], the Paleolithic archaeological evidence is not clearly visible to understand peopling of this country [22]. Nonetheless, the Indus Valley and Harappan civilizations portray footprints of Neolithic period [23] suggestive of the arrival of Indo-European speakers who established the caste system, an anthropologically significant prehistoric event [24,25]. The cultural and historical importance of the arrival and settlement of the Indo-Aryans is undisputed, but it is not clear if this was established through 'replacement of the existing people by outsiders' [22] or did the 'people already in India changed their habits and cultures?' [22]. Such questions have never been addressed in an unambiguous manner, even though the potential of polymorphic DNA markers in reconstruction of human migration and phylogeography [26,27] has long been appreciated. It appears that even carefully planned geographic genomics studies remained largely speculative due to the lack of a universal 'gold standard' as the classical mitochondrial DNA markers offer too few informative polymorphisms and the newly developed Y – chromosome markers are even less polymorphic than mitochondrial hypervariable regions [2]. Lately, new genetic models were successfully harnessed based on parasites and pathogens that probably accompanied their human host during evolution and much of the human history including migrations and expansions [2,4,5] in different continents. Such approaches constitute an attractive alternative to reconstruct human origins and spreads, population dynamics and bottlenecks, wars and displacements, farming and plagues etc.

Our study was aimed at tracking ancient origins of the Indian *H. pylori *through a two-pronged approach to i) substantiate European link of the pathogen in India and ii) to prove that the pathogenicity island was also of European origin and this PAI has not been a 'recent' addition to the genome of Indian *H. pylori*. Our analyses, based on MLST and comprehensive genotyping of the *cag*PAI, linked about 100% of the Indian isolates to *H. pylori *sub-population hpEurope. This perhaps conveys the message that *H. pylori *was most probably introduced to the Indian subcontinent by ancient Indo-European nomadic people and our findings, therefore, are consistent with the idea of a possible gene flow into India with the arrival of Indo-Aryans.

Overall, based on the MLST data (Figure 2) and the *cag*PAI patterns (Figure 3), we suggest that *H. pylori *might have arrived in India probably at the same time when Indo-European language speaking people crossed into India (~4000–10,000 years before present). Alternatively, the unquestionable common origin of Indian strains with the European ones could be actually more ancient, following the upper Paleolithic spread of *Homo sapiens *in Eurasia, as suggested by mtDNA variability [18], and our data on *H. pylori *MLST do not rule out this possibility.

Present day India represents a 'genetic playground' with tremendous diversity of cultures, and languages. However, the people are largely stratified as tribals and nontribals [25]. Four main language families are spoken, the largest being, Indo European (IE), which is prevalent in North, and the second largest Dravidian (DR) group represents languages spoken in the South [28]. The other two language groups include Tibeto-Burman (TB) of the Sino-Tibetan and the Austro-Asiatic (AA) families, largely spoken in far North and the North-east India. While most of the IE speakers belong to castes, the majority of the tribal communities (>450) speak about 750 different dialects that fit within any one of the other three language families (DR, TB, AA) [25,28]. Such an enormous cultural diversity might argue for many different populations and sub-populations of *H. pylori*. But until now, and including this study, *H. pylori *with genetic features of hpEurope have only been reported from India [29,30]. Even the newly described sub-population hpAsia2 from Ladakh is also a variant of hpEurope and many Ladakhi strains that we looked at in this study, clustered with European *H. pylori *clade (Figure 2). Also, the *cag*A sequences from *H. pylori *belonging to tribal Oraon and Santhals were indistinguishable from those of the mainstream Indians and Europeans (Figure 4), indicating sweeping spread of a single *H. pylori *genotype across the Indian peninsula. Moreover, we did not document presence of any other *H. pylori *populations and sub-populations such as hspAmerind, hspMaori, hpAfrica and hpEast Asia in the limited, but representative culture collection that we looked at. However, the visible footprints of other migrations into India such as from the North Eastern corridor and the presence of phenotypic features resembling to Africans in the South, make it unwise to presume an '*H. pylori *free India' at the time of arrival of Indo-European speaking invaders. This issue and the fact that *H. pylori'*s first association with humans traces back to millions of years before present, in Africa [6,17], it is more realistic to hypothesize that *H. pylori *of African and Asian gene pool might have already been present in India. The predominance of a single *H. pylori *population might therefore, point to a distinct survival advantage conferred by a fully functional (western type) *cag*PAI. This analogy is consistent with the scenario we previously reported [3] for the South American, Amerindian strains, which were presumably out competed by their Spanish counterparts arriving with an intact and functional western *cag*PAI.

Finally, it is possible that phylogeny based on highly recombining gene loci [15,29,31-35] may not be completely foolproof to extract inheritance from different ancestral populations, especially when we use tools such as MEGA 3.0 [36], which do not support admixture analysis. Moreover, phylogenetic methods based on bifurcating trees, such as Neighbor joining analysis, may not be fully appropriate for analysis at the intra-species level [37,38], especially in case of hypervariable genomic regions, where multiple homoplasy due to reversions, recurrent mutations etc., or polytomy may sometimes confound the phylogenetic interpretation. However, the housekeeping genes used here are selectively neutral and uniform as compared to virulence associated loci such as the flagellins and *vac*A [10], and therefore, recombinant and hybrid alleles that blur lineage inferences, could be a rare occurrence and not a routine. Partly in view of this assumption and due to our previous experiences on dissecting complex ancestry of native Peruvian isolates using phylogenetic methods [3] we did not attempt admixture analysis with complicated Bayesian statistics. However, to ensure that our conclusions did not represent shortcomings of a single method, we adopted an integrated phylogenetic approach combining MEGA/NETWORK based analyses and genotyping strategy based on full *cag*PAI and its left and right end sequences. Interestingly, these approaches unambiguously show the Indian *H. pylori *genotypes scattered among the European ones. Although this would be consistent with gene flow into India with the Indo-Aryans, or even more ancient origins following the Paleolithic expansion of humans in Eurasia, but also consistent with another scenario: migration from India to Europe. However, the later scenario becomes insignificant due to the unavailability of supporting archeological, linguistic and historical data. Nonetheless, an understanding of the time-scale would be helpful for choosing between such explanations, with the estimation of divergence times between the *H. pylori *sequences in the different human populations. These issues therefore need to be addressed in future.

## Conclusion

In summary, we found significant overlap among genetic identities of Indian and European *H. pylori *based on core and flexible genome markers. This remarkable genetic similarity points to their possible common genetic origins and could therefore be potentially useful in understanding entry, survival, spread and adaptation of *H. pylori *in Indian stomachs. Also, this study is consistent with the hypothesis of co-evolution of *H. pylori *with *H. sapiens *and therefore, could form a reliable foundation to test and reconstruct gene flow into India with the arrival of Indo-Aryans or otherwise.

## Methods

### Bacterial strains, genomic DNA and diagnostic PCR

All the strains were cultured by the Centre for Liver Research and Diagnostics, Deccan college of Medical Sciences, Hyderabad, from patient biopsies. All the biopsy material was collected with necessary ethical clearances and after obtaining informed consents. Template DNA was prepared from single colony picks as described previously [39]. Genomic DNAs of the 10 Ladakhi strains were received from Mark Achtman, Max-Planck Institute für Infektionsbiologie, Berlin, Germany. Genomic DNA was isolated from strains obtained from patients with different disease types including Duodenal Ulcer (DU); Gastric Ulcer (GU); Gastric Cancer (GC); Gastritis (G); Non Ulcer Dyspepsia (NUD); Peptic Ulcer Disease (PUD); Chronic Duodenal Ulcer (CDU); Portal Hyper Tension (PHT) etc. (Figure 1). However, in the current study, the clinical background of the individual isolates was not taken into account. The Indian isolates we looked at (n = 63) were originally from Native Indian people mainly of Aryan and Dravidian ancestry from India. PCR based analyses of genes namely *cag*A, *glm*M, *bab*B [14] and *oip*A were carried out to ascertain the quality of DNA samples we used. Also these PCR assays served as amplification level controls for the analysis of insertion, deletion and substitution in the *cag*PAI.

### MLST analysis by MEGA 3.1 and NETWORK 4.2.0

A 600 bp region each from the 7 housekeeping genes spread throughout the genome *atp*A, *efp*, *ure*I, *ppa *and *mut*Y, *trp*C, *yph*C was amplified by PCR and sequenced for all the Indian isolates exactly as described previously [3]. Sequencing was performed on both the strands, using an ABI Prism 3100 DNA sequencer (Applied Biosystems, USA). PCR and direct sequencing were performed at least twice to determine and confirm the DNA sequences for each isolate. Consensus sequence for each of the samples was generated using Genedoc (version 2.6.002). Multiple alignments of sequenced nucleotides were carried out using Clustal X (version 1.81). Neighbor joining trees were constructed in MEGA 3.0 [36] using bootstrapping at 10000 bootstrap trials and through Kimura-2 parameters. For beginning construction of phylogenetic trees based on MLST genotyping procedures, ~400 sequences of the 7 housekeeping genes of strains belonging to different established genotypes, including 40 sequences of isolates from Ladakh were obtained from the pubMLST database [40] (courtesy, Daniel Falush). The Indian *H. pylori *diversity represented in the final MEGA3.0 alignment and the tree thereof comprised of a total of 63 sequences inclusive of the 10 Ladakhi sequences generated in house along with the other 9 representative Ladakhi sequences from the database. We performed on MLST sequence data a network analysis using the program Network 4.2.0.0. [38,41]. In particular, the median-joining algorithm for multistate DNA data was used [42,43]. Because of a program limitation, which cannot handle more than 1000 polymorphic sites at once, we performed the analysis separately on two halves of the sequence (encompassing respectively 650 and 665 polymorphic sites). The input file (in *.rdf format) was obtained using the commercial software DNA Alignment 1.1.2.1.

### Profiling of the *cag*A gene, the whole *cag*PAI and its right junction

The 5' end of the *cag*A gene was amplified using primers mentioned elsewhere [44] and the amplified products were sequenced with forward and reverse primers. The consensus sequences were then translated into amino acid sequences using GeneDoc software (version 2.6.002) and were then assigned to the Western or the East Asian group based on the C or D repeats present respectively in the EPIYA motif [45]. Genetic diversity of the *cag*A 5' end sequences for our Indian isolates: MS15, MS7, MS4 and 3K along with 26695 and J99 were compared to the other records from GenBank [20,30,46]. A phylogenetic neighbor-joining tree was constructed by MEGA 3.1 version using these sequences (Figure 4).

PCR analyses were carried out to find the status of the *cag*PAI using 8 sets of primers that amplified the *cag*A gene, its promoter region, the *cag*E and *cag*T genes and the left end of the *cag*PAI [8,29,34]. We also analyzed whole *cag*PAI of the representative isolates from India (3K, 4K, 3C, MS40 and MS38) by PCR using overlapping primers as described by Blomstergren and colleagues [9]. The entire *cag*PAI sequence of a single representative Indian isolate 3K was determined. The complete *cag*PAI sequence was aligned by VISTA programme [47] against other PAI sequences belonging to strains 26695, J99, HPAG1 and 13 other clinical isolates corresponding to *H. pylori *sub-populations hpEurope, hpEast Asia and hpAfrica1 (Figure 3B).

Chromosomal rearrangements are known to give rise to 5 types of insertion-deletion and substitution motifs in the region between the right end of *cag*A gene and the glutamate racemase (*glr*) gene (*cag*-RJ). We assessed these rearrangement profiles for all of the Indian isolates by PCR as described earlier by Kersulyte and colleagues [13].

### Analysis of the chromosomal plasticity region cluster

Chromosomal plasticity region ORF's were assessed for all the 63 Indian isolates by PCR based typing to ensure that all the strains that we looked at were independent and non-clonal by descent. The PCR primers and the procedures used for evaluating the presence of the plasticity region ORF's (JHP912, HP986, JHP947, JHP926, JHP944, JHP931, JHP945 and JHP933) have been descried previously [48].

### Nucleotide sequence accession numbers

The nucleotide sequences of the 7 housekeeping genes for the 23 representative Indian isolates have been deposited in the GenBank [Accession numbers, GenBank: DQ504165–DQ504183 and DQ927245–DQ927248 (*atpA*), DQ504184–DQ504202 and DQ927249–DQ927252 (*efp*), DQ504203–DQ504221 and DA927253–DA927256 (*mutY*), DQ504222–DQ504240 and DQ927257–DQ927260 (*ppa*), DQ504241–DQ504259 and DQ927261–DQ927264 (*trpC*), DQ504260–DQ504278 and DQ927265–DQ927268 (*ureI*), DQ504279–DQ504297 and DQ927269–DQ927272 (*yphC*)]. These sequences will also be made available through the pubMLST database maintained at the Max-Planck Institute für Infektionsbiologie, Berlin, Germany. The sequence of whole *cag*PAIs of the representative Indian isolate 3K and the French isolate Fr908 for which the sequence was determined in our laboratory, have been deposited in Genbank under accession nos. DQ985738 and EF195721 respectively. These and other sequences can also be requested from the authors.

## Authors' contributions

SMD and IA performed and analyzed MLST, all other genotyping experiments and phylogenetic analysis. SMD also helped in analysis of *bab*B and *oip*A genotyping. MAA performed *vac*A genotyping. IA also performed *H. pylori *isolation and culture. YA carried out *in silico *analysis of the *cag*PAI sequences. PF performed Network analysis on MLST data and contributed to manuscript writing. LAS and FM provided expert clinical and epidemiological support and contributed to discussions and manuscript writing. NA planned and supervised the study, edited the final draft of the manuscript and provided overall leadership. All the authors read and approved the final manuscript.
